# Applying the UK standards for public involvement: reflections and lessons from case studies across the UK

**DOI:** 10.1186/s40900-026-00967-5

**Published:** 2026-09-10

**Authors:** Alan McMichael, Janet Diffin, Barbara Molony-Oates, Peter Gee, Bob MacAllister, Zoe Gray, Alex Newberry, Eleanor Jackson, Angela Polanco, Una Rennard, Julie Simpson, Gwynneth Cracknell

**Affiliations:** 1 Health and Social Care, Research & Development, 9th Floor Linum Chambers, Bedford Street, Belfast, BT2 7ES UK; 2 Health Research Authority, London, UK; 3 Health and Care Research Wales, Cardiff, UK; 4 Health and Care Research, Wales Public Involvement Delivery Board, Cardiff, UK; 5 National Institute for Health and Care Research, London, UK; 6 NIHR INVOLVE, Southampton, UK; 7 Chief Scientist Office, Edinburgh, Scotland

**Keywords:** Public involvement, UK standards for public involvement, Involvement in research

## Abstract

**Background:**

Public involvement in health and social care research improves relevance, transparency, and impact. Since their launch in 2018, the UK Standards for Public Involvement have provided a practical, values-based framework to support meaningful public involvement across all stages of research.

**Methods:**

This Comment article draws on three applied case examples at national, regional, and study-level to illustrate how the Standards are being implemented across the UK. The authors reflect on lessons learned through implementation and identify emerging challenges and opportunities for embedding the Standards in evolving research contexts.

**Results:**

The UK Standards for Public Involvement have been applied to evaluate public involvement quality in NIHR research centres, to assess the role of contributors in clinical trials, and to inform feedback, practice, and collaboration. The Standards underpin the Shared Commitment to Public Involvement and have contributed to shifting public involvement from aspiration to expectation, providing a consistent reference point for improvement and accountability.

**Conclusions:**

The UK Standards for Public Involvement promote inclusive, ethical, and impactful involvement. We call on the health and social care research community to promote, apply, and build upon the UK Standards for Public Involvement to support high-quality public involvement into the future.

## Abstract (plain language summary)

When people with lived experience, such as patients, carers, and members of the public are involved in health and care research, the research is more likely to be relevant, transparent, and useful. In the UK, a set of guidelines called the UK Standards for Public Involvement were introduced in 2018 to help researchers and the public work better together.

The Standards describe what good public involvement should look like, and encourage learning and reflection. They have been used across the UK to improve how research teams plan, carry out, and evaluate public involvement. For example, the Standards have been used to assess research reports, support feedback to public contributors, and develop new tools and resources.

This editorial explains how the UK Standards have helped promote a stronger culture of involvement in research and continue to offer a practical and flexible framework for making sure that public involvement is meaningful, inclusive, and ethical.

We encourage everyone involved in research including researchers, funders, public contributors, and policy-makers, to keep using and promoting the UK Standards so that public involvement remains a vital part of research in the UK.

## Introduction

Public involvement refers to how patients, carers, and members of the public, can work in partnership with researchers to shape the decisions made about research [1]. It is more than simply consulting with patients or engaging with the public to share research findings. This approach recognises that people’s lived experience offers valuable insights that can enhance the quality, relevance, and impact of research [2].

Involving the public in research is both a moral imperative and a practical necessity. It improves transparency, fosters trust, and ensures that research asks the right questions and reaches the right people. These benefits have been well documented [3], and public involvement is now routinely required by research funders, regulators, and ethical review boards across the UK.

## The UK Standards for Public Involvement

**Fig. 1:**
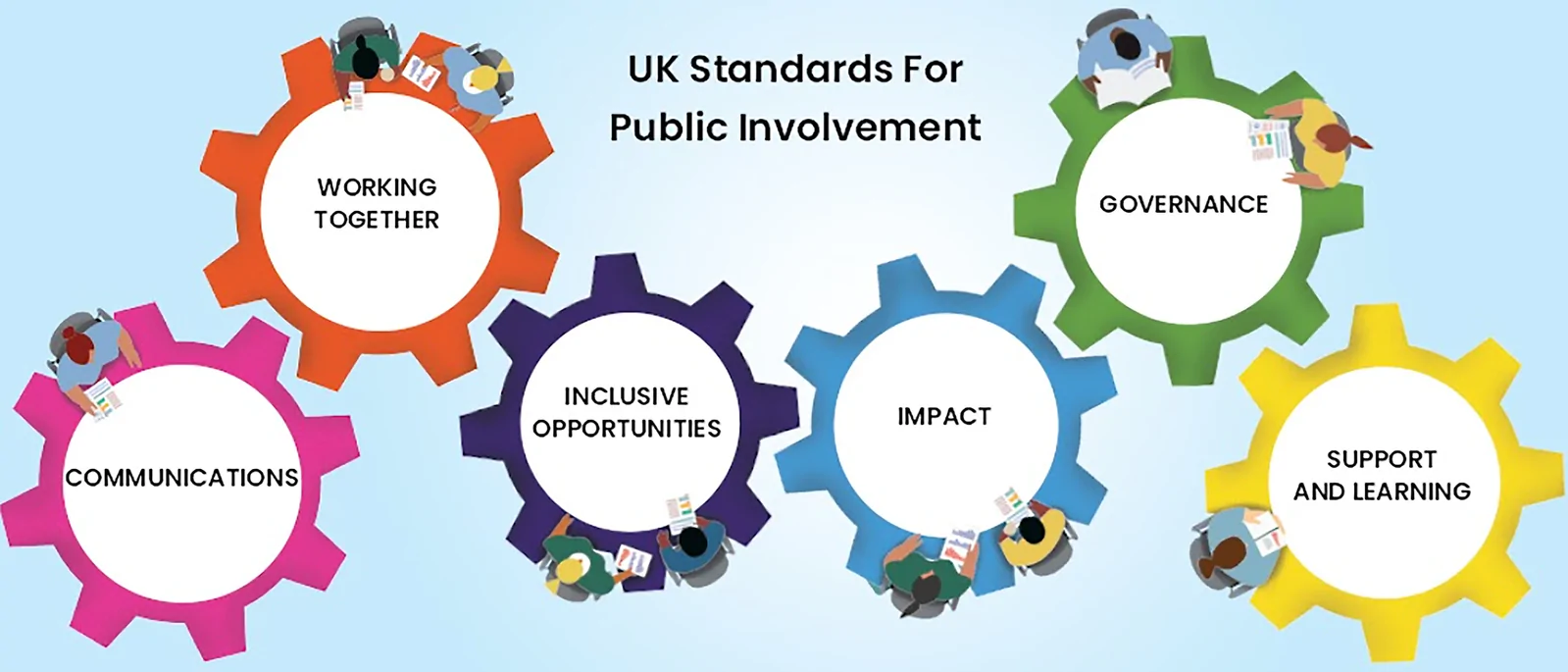
The 6 UK Standards for public involvement

In 2015, the National Institute for Health and Care Research (NIHR) strategic review of public involvement recommended the creation of co-produced, practical standards with self-assessment tools. At the same time within Wales, standards for public involvement were initiated by Health and Care Research Wales’ Public Delivery Board [4]. Subsequently, in 2016, a multi-stakeholder workshop in 2016 between England, Scotland, Wales and Northern Ireland helped shape the next steps which emphasised the need for structured principles and guidance across organisations for public involvement. After an early development phase and a public consultation on the UK Standards in 2017, the UK Standards for Public Involvement were officially launched in March 2018 [5]. The UK Standards were designed to be used as a framework for what good public involvement in research looks like and encourage reflection and learning [5]. The UK Standards for Public Involvement are built on six standards which are summarised in Fig. 1 and defined in Table 1. Since the development of the UK Standards, a Five nations group was formed to have guardianship of and a UK Standards for Public Involvement. The UK Standards continue to be discussed and shared through the quarterly 5 Nations PPI meeting, a collaborative forum involving public involvement leads from England, Scotland, Wales, Northern Ireland, and the Republic of Ireland. The group provides an opportunity for shared learning, discussion of emerging challenges, and exchange of good practice relating to public involvement in Health and Social Care research.

Below, we present three illustrative case studies demonstrating how the UK Standards for Public Involvement have been applied across organisational, regional, and project-level contexts. We also reflect on the wider influence of the Standards through national initiatives that have helped promote and embed their principles across the health and social care research landscape. This commentary does not seek to provide a systematic review of all applications of the UK Standards for Public Involvement, nor to comprehensively evaluate their impact. Rather, it draws on a series of illustrative case examples identified by the authors to highlight different ways in which the Standards have been applied across research, organisational, and regional contexts. Through these examples, we explore the practical utility of the Standards, reflect on lessons learned from their implementation, and consider their continuing role in supporting meaningful public involvement in research.

## The application of the UK Standards for Public Involvement

Since their publication, the UK Standards for Public Involvement have been used in a variety of ways across organisations, regions, and studies. The examples presented in this commentary were identified through discussions among the co-authors and were purposively selected to illustrate different ways in which the UK Standards for Public Involvement have been applied across national, regional, and study-level contexts. The examples were not intended to provide a comprehensive review of all applications of the Standards, but rather to highlight practical experiences, learning, and reflections arising from their use in different settings.

Moult et al. [6] used the UK Standards for Public Involvement as part of a framework documentary analysis of 112 annual reports submitted by NIHR-funded research centres. The six Standards provided a structured framework through which reported public involvement activities could be assessed, while an accompanying quality improvement framework was used to explore the quality of involvement practices. The evaluation identified examples of activity across all six Standards and highlighted both areas of good practice and opportunities for further development. Importantly, the study demonstrated how the Standards can be applied beyond planning and delivery to support reflection, evaluation, and organisational learning. The findings also suggested that there is no single way of meeting the Standards, with implementation varying according to local context, available resources, and organisational priorities. This highlights both the flexibility of the Standards and the importance of adapting them to different research environments.

Seddon et al. [7] conducted the first retrospective evaluation of a multinational clinical study using the UK Standards for Public Involvement as an evaluation framework. The authors examined how public contributors had been involved throughout the design, delivery, and dissemination of the Hospice In-patient Deep Vein Thrombosis Detection (HIDDen) study, drawing on interviews, field notes, and a purpose-designed review tool informed by the six Standards. The evaluation found evidence of activity across all six Standards, with particular strengths relating to ‘Working Together’ and ‘Support and Learning’. Public contributors influenced key aspects of the study, including participant information materials, recruitment approaches, and decisions relating to proxy consent. The retrospective review also identified areas where involvement could have been strengthened, particularly in relation to governance and leadership. Importantly, the authors concluded that the greatest value of the Standards may be realised when they are considered from the outset of a project rather than applied retrospectively. This case study demonstrates how the Standards can support structured reflection on public involvement activities, helping research teams identify both successful approaches and opportunities for future improvement.

At a regional level, Mathie et al. [8] used the UK Standards for Public Involvement as a framework for a retrospective evaluation of a collaborative research project involving researchers, PPI contributors, and PPI leads from across the East of England. The study explored how a regional network supported the design, delivery, and dissemination of research examining feedback to public contributors. Drawing on meeting evaluations, reflective discussions, correspondence, and contributor reflections, the Standards were used to structure a detailed assessment of what worked well and where challenges were encountered. The evaluation highlighted several benefits of regional working, including access to established networks, opportunities for shared learning, stronger communication pathways, and the ability to bring together diverse perspectives from researchers and public contributors. However, the Standards also helped identify practical barriers to effective involvement, including difficulties involving contributors before research funding was secured, navigating different organisational payment processes, and balancing support needs across geographically dispersed groups. Importantly, the authors concluded that the Standards provided a useful framework for identifying both strengths and gaps in involvement practice, supporting reflection, organisational learning, and future improvement. The study also highlighted that implementation of the Standards is often influenced by real-world organisational and administrative constraints, reinforcing the importance of flexibility when applying the framework in different settings.

Beyond individual projects and organisations, the influence of the UK Standards can also be seen at a strategic and system-wide level through the Shared Commitment to Public Involvement [9]. Developed collaboratively by major health and social care research organisations across the UK, the Shared Commitment brings together funders, regulators, research organisations, and public involvement leaders around a shared ambition to improve the quality, consistency, and inclusivity of public involvement in research. The Commitment explicitly references the UK Standards for Public Involvement as a framework for excellent involvement practice and includes a commitment to promote their use across the research sector. Through its focus on learning, inclusion, evidence of impact, and organisational accountability, the Shared Commitment reflects many of the principles underpinning the Standards. This example illustrates how the Standards have influenced not only individual research projects and evaluations, but also wider strategic approaches to public involvement. Importantly, the Shared Commitment demonstrates the value of the Standards as a shared framework and common language that can help align organisations around collective goals for improving public involvement across the UK research landscape. While formal evaluation of the Shared Commitment is still emerging, its adoption by a large number of over 30 nationwide partner organisations demonstrates growing recognition of the importance of coordinated approaches to public involvement.

## Discussion

The case studies presented suggest that the UK Standards provide a practical framework for planning, delivering and reflecting on public involvement activities. These research examples, along with other published research [10, 11], demonstrate how the Standards are not just aspirational, they are being used actively to evaluate, strengthen, and evolve practice. Furthermore, emerging tools like the Public Involvement in Research Impact Toolkit (PIRIT) [12], a resource for planning and demonstrating the impact of public involvement, and the Health Research Authority’s work on people-centered research, illustrate the growing ecosystem of support for involvement that aligns closely with, and are built upon the Standards. Moreover, other initiatives such as The Public Engagement in Data Research Initiative (PEDRI), were influenced significantly by the UK Standards for Public Involvement [13].

As research priorities shift towards more inclusive, community-based models, greater use of secondary data, and expanding commercial involvement, the UK Standards for Public Involvement remain highly relevant. The principles of inclusive opportunities and governance are particularly crucial in light of the NIHR current strategic commitment to Public Involvement [4], and the O’Shaughnessy Review into commercial clinical trials [14] which echoed that more diverse participation in research relies on excellent public involvement. It is critical that future research appropriately measures the impact of public involvement and promotes inclusive partnerships as illustrated by the UK Standards for Public Involvement [5]. At a time when public involvement is expected, but not always supported or evaluated systematically, the Standards provide both clarity and consistency. Taken together, these examples suggest that the UK Standards can be adapted to different contexts while maintaining a best practice framework for Public Involvement. The examples also highlight areas where further evidence development would be valuable, including systematic evaluation of the Standards’ impact. Nevertheless, as practice continues to evolve, these case studies provide useful insights for how the Standards can inform be used more widely and inform future initiatives.

However, while the UK Standards for Public Involvement provide an important shared framework for supporting and evaluating involvement practice, questions remain regarding how the Standards are interpreted, implemented, and evaluated across different contexts. In some settings, the Standards may support reflection, learning, and improvement, while in others there is a risk they may be applied primarily as benchmarking or assurance tools. As public involvement practice continues to evolve, there is therefore a need not only to promote the Standards, but also to critically reflect on how they are used in practice and how they may continue to adapt to changing research landscapes. Our overview of how the UK Standards for Public Involvement highlight how they can be inconsistently applied. For instance, in three of the case studies [6–8] the standards were applied retrospectively, despite the fact that the standards are also suitable for planning public involvement throughout a research project or in Health and Social Care service development.

## Conclusion: Staying the course, raising the bar

The UK Standards for Public Involvement set clear expectations on how to ensure that public involvement is of high quality, whilst considering the principles partnership working, transparency, and respect. The case studies presented illustrate how the UK Standards have been applied in a range of research settings, however further evaluation is recommended to understand the longer-term impacts on research practice, organisational culture and public involvement across the UK.

It was once said that ‘the rising tide lifts all boats,’ therefore, as we look ahead, it is essential not to “reinvent the wheel,” but rather, to build on the firm foundation the Standards initially provided. Moving forward, we call on researchers, policymakers, public involvement leads, and public contributors to actively promote and apply the UK Standards for Public Involvement, embedding them into the health and social care landscape not just as a tool for improvement, but as a reflection of our shared commitment to excellent, ethical, and inclusive research. Looking ahead, there is an opportunity for the research community to build a more explicit evidence base around the Standards, complementing their current values-based foundation.

**Table 1:** Overview of Each of the UK standards for public involvement

Standard	Definition
**Inclusive opportunities**	Public involvement partnerships are accessible and include a range of people and groups, as informed by community and research needs.
**Working together**	Work together in a way that values all contributions, and that builds and sustains mutually respectful and productive relationships.
**Support and learning**	Offer and promote support and learning opportunities that build confidence and skills for public involvement in research.
**Governance**	Offer and promote support and learning opportunities that build confidence and skills for public involvement in research.
**Communications**	Use plain language for well-timed and relevant communications, as part of involvement plans and activities.
**Impact**	Seek improvement by identifying and sharing the difference that public involvement makes to research.

## Data Availability

No datasets were generated or analysed during the current study.
